# PD27 - Specific antibodies in oral immunotherapy for cow’s milk allergy: kinetics and prediction of clinical outcome

**DOI:** 10.1186/2045-7022-4-S1-P27

**Published:** 2014-02-28

**Authors:** Emma M Savilahti, Mikael Kuitunen, Mika Mäkelä, Erkki Savilahti

**Affiliations:** 1 Hospital for Children and Adolescents, University of Helsinki, Helsinki, Finland; 2 Hospital for Allergic and Skin Diseases, University of Helsinki, Helsinki, Finland

## Background

Specific antibodies to cow’s milk (CM) may have prognostic value in CM allergy. We hypothesized that they may also help in predicting the clinical outcome of oral immunotherapy (OIT) prior to treatment, and that changes in specific antibody levels during the therapy may reflect its efficacy.

## Methods

We investigated 40 children aged 6-17 year with cow’s milk allergy who either successfully completed OIT (n=32) or discontinued the therapy due to side effects (n=8). We analyzed in sera drawn before and after OIT specific IgA, IgG, IgG1 and IgG4 to CM, casein, beta-lactoglobulin and ovalbumin with enzyme linked immunosorbent assay, and IgE to CM and hen’s egg white with enzymatic fluoroimmunoassay (Pharmacia CAP system).

## Results

Specific IgA, IgG, IgG1 and IgG4 to CM and casein, and CM specific IgE prior to OIT were higher in children who eventually discontinued the therapy compared with children who achieved desensitization (p<0.05). Side effects in the entire population were associated with high IgG, IgG1, but low IgG4 levels to ovalbumin (p<0.05). Specific IgA, IgG, IgG1 and IgG4 to CM proteins significantly increased and IgE to CM decreased from the start to end of OIT in children who achieved desensitization (p<0.01), whereas in those who interrupted OIT only IgG, IgG1 and IgG4 to CM increased significantly (p<0.03).

## Conclusions

High specific IgE, IgA and class IgG antibodies to CM proteins appear to predict failure to achieve desensitization in CM OIT. Specific IgA and class IgG antibodies to CM increase and CM IgE decreases during desensitization.

